# Gendered Body Mass Index Percentile Charts and Transgender Youth: Making the Case to Change Charts

**DOI:** 10.1089/trgh.2019.0016

**Published:** 2019-10-29

**Authors:** Kacie M. Kidd, Gina M. Sequeira, Cherie Priya Dhar, Gerald T. Montano, Selma Feldman Witchel, Dana Rofey

**Affiliations:** ^1^ Center for Adolescent and Young Adult Health, UPMC Children's Hospital of Pittsburgh, Pittsburgh, Pennsylvania.; ^2^ Ann & Robert H. Lurie Children's Hospital of Chicago, Northwestern University Feinberg School of Medicine, Chicago, Illinois.; ^3^ Division of Pediatric Endocrinology, UPMC Children's Hospital of Pittsburgh, Pittsburgh, Pennsylvania.; ^4^ Department of Psychiatry, Pediatrics, Psychology, University of Pittsburgh School of Medicine, Pittsburgh, Pennsylvania.

**Keywords:** adolescence, clinical research, obesity/overweight, transgender

## Abstract

Body mass index (BMI) is defined as weight (kg)/height^2^ (m^2^). Differences in BMI percentiles between sexes confound the diagnosis of weight-related disorders in transgender youth because choosing the appropriate chart is challenging. Data on BMI measures are needed for transgender youth, but there are no guidelines on how to collect or report this data. We use two theoretical cases to assert that health care providers and researchers should consider use of both male and female growth charts for transgender youth, particularly for individuals at the extremes of weight.

## Introduction

**FIG. 1.**
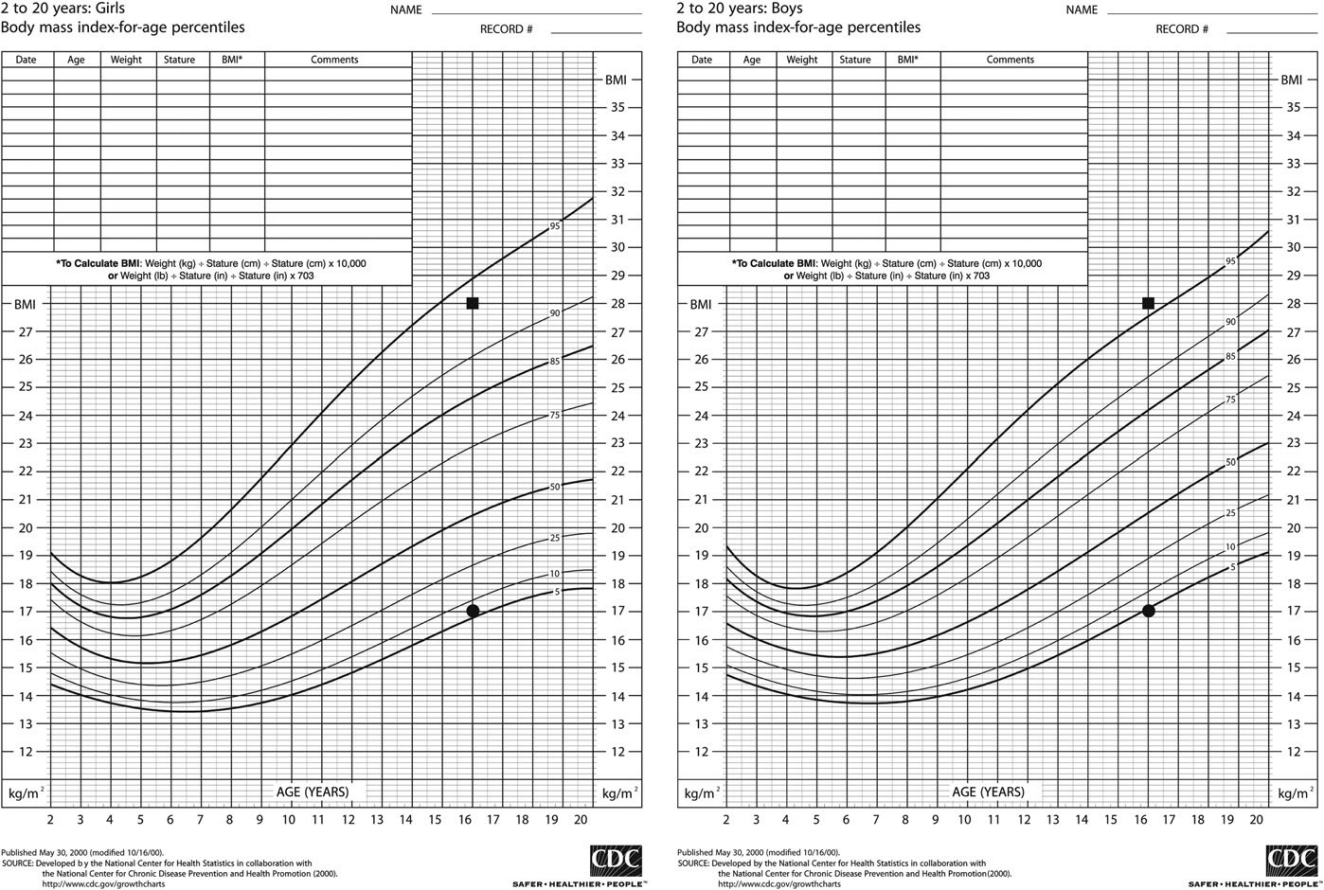
Gendered BMI percentile growth charts from CDC.gov. The patient from Case 1 (Sam) has a single time point measurement represented by the squares and the patient from Case 2 (Lea) has a single time point measurement represented by the circles. BMI, body mass index.

Growth charts were developed by the Centers for Disease Control for use in the United States in 1977, and were subsequently revised in 2000 using data from the National Health and Nutrition Examination Survey (NHANES).^1^ The update included body mass index for age (BMI percentile) charts for male and female children to better characterize body fat.^1^ Health care providers use BMI percentile charts to identify overweight and underweight children by their overall growth trends. Subsequently, these children can be evaluated to assess their need for behavioral or medical weight-related interventions. The charts differ in the range of expected norms between male and female children with the female chart having a wider span at the extremes of weight (Fig. 1).^1^

Available data regarding height, weight, and BMI trends in transgender youth are sparse, and the extent of pubertal blockade with gonadotropin-releasing hormone (GnRH) analog therapy in these patients is unclear.^2–4^ Studies in adults suggest that testosterone not only increases muscle mass but is also associated with abdominal obesity and a modest increase in BMI, whereas estradiol is associated with a lower waist to hip ratio due to fat deposition in the hips.^5,6^ In some instances, puberty blocking medications are used to promote linear growth by delaying skeletal maturation, further complicating the relationship between height and weight.^7^ Recommendations regarding initiation of GnRH analogs and gender-affirming hormones vary. Current guidelines emphasize that young people should achieve pubertal development concordant with peers. How these individualized timelines will impact linear growth or BMI is unknown.^8,9^ As an example of the potential complexity of using gendered BMI percentile growth charts, we describe two theoretical cases that illustrate the need to consider using growth charts that correspond to both affirmed gender and sex assigned at birth.

## Case 1

Sam is a transmasculine patient (assigned female at birth and identifies as male). This patient was started on a GnRH analog (puberty blocker) at Tanner (Sexual Maturity Rating, SMR) 2 and continued on the puberty blocker until after starting testosterone at age 15 years. At age 16 years he was found to have a BMI of 28 kg/m^2^. Sam's gender marker has not been legally changed; therefore, the electronic medical record (EMR) at his provider's office automatically denotes him as female. Per the BMI percentile growth chart for females, he is considered to be “overweight” (94th percentile). Given his pubertal hormone exposure and subsequent growth more closely resembling a cisgender male, one could argue that he should be plotted on the male BMI percentile curve. Per the growth chart for males, his BMI percentile is considered to be “obese” (96th percentile).

## Case 2

Lea is a transfeminine patient (assigned male at birth and identifies as female). This patient presented for gender care at Tanner (SMR) 5 and did not receive a GnRH analog for pubertal blockade. Her gender marker was legally changed so the EMR automatically plots her on the female BMI percentile growth chart. Her BMI at age 16 years is 17 kg/m^2^. On the female growth chart she is considered normal weight (sixth percentile). One could argue that since her pubertal hormone exposure and subsequent growth more closely resembles a cisgender male, she should be plotted on the male BMI percentile curve and per this growth chart, her BMI percentile is considered to be “underweight” (fourth percentile).

## Conclusion

In both cases, providers may fail to recognize the need for these patients to receive appropriate interventions for weight. In addition to health concerns for individual patients, evidence-based recommendations for the use of gendered BMI percentile curves for outcomes research is lacking. As this research lays the groundwork for future weight-based interventions, understanding how to adapt the use of these measures for this population is essential. Both gender care specialists and primary care providers should think critically in deciding which growth charts are optimal for individual patients based on prior patient history, pubertal stage, and any gender-affirming pharmacological interventions that may alter height/weight. When necessary, they should also consult with multidisciplinary teams to ensure that other medical concerns are not overlooked. Research methods that accurately define the BMI status of transgender patients need to be developed, and standardization of these methods may help with obtaining long-term patient outcomes regarding cardiovascular disease, diabetes, osteoporosis, and other conditions associated with BMI abnormalities.

Providers should be aware of the role of the EMR in determining the growth charts used and consider the potential value of trending the patient on both male and female charts, particularly when the patient is at the extremes of normal weight. We propose that dual trending should be considered in all gender diverse youth. This approach may help navigate the inherent limitations of using gendered growth charts for youth who identify as nonbinary. Ultimately, additional investigations are needed to fully understand the impact of GnRH analogs and gender-affirming sex steroid hormone treatment on weight and linear growth trends in transgender youth. Availability of these data has the potential to inform patient-centered interventions and to improve health outcomes.

## Abbreviations Used

BMI: body mass index
EMR: electronic medical record
NHANES: National Health and Nutrition Examination Survey
SMR: Sexual Maturity Rating
